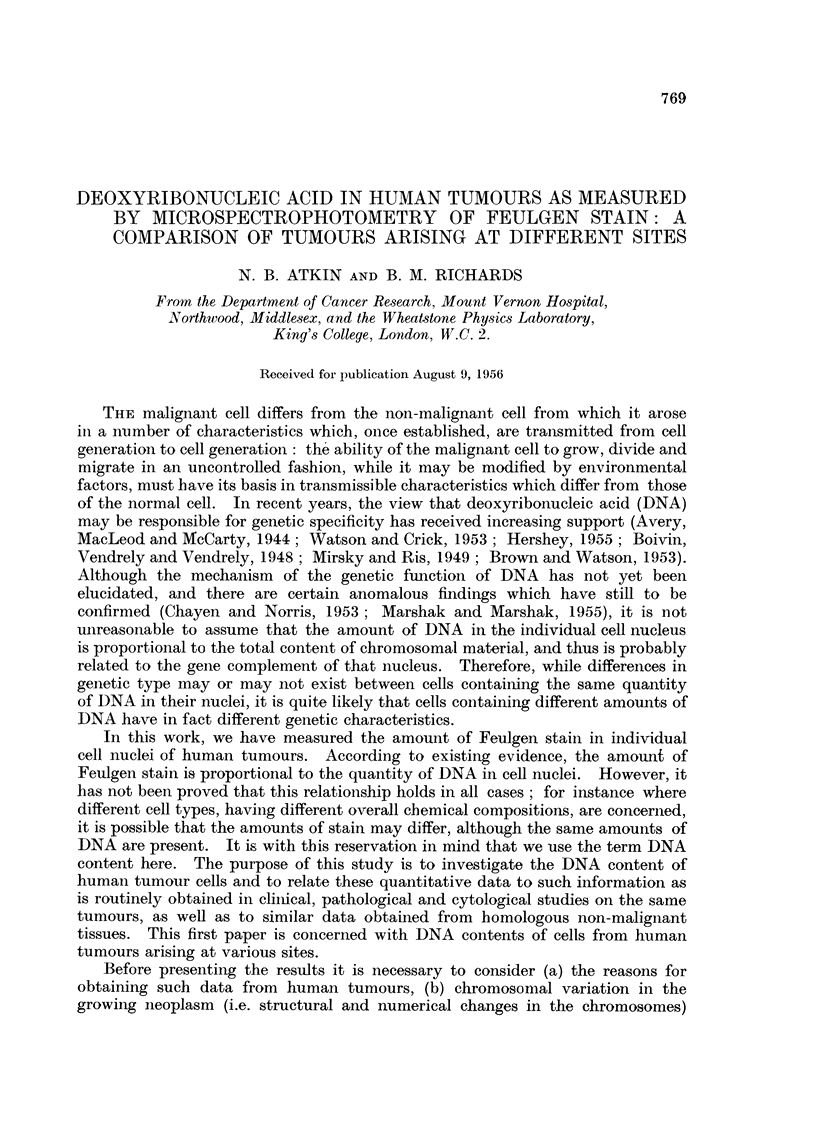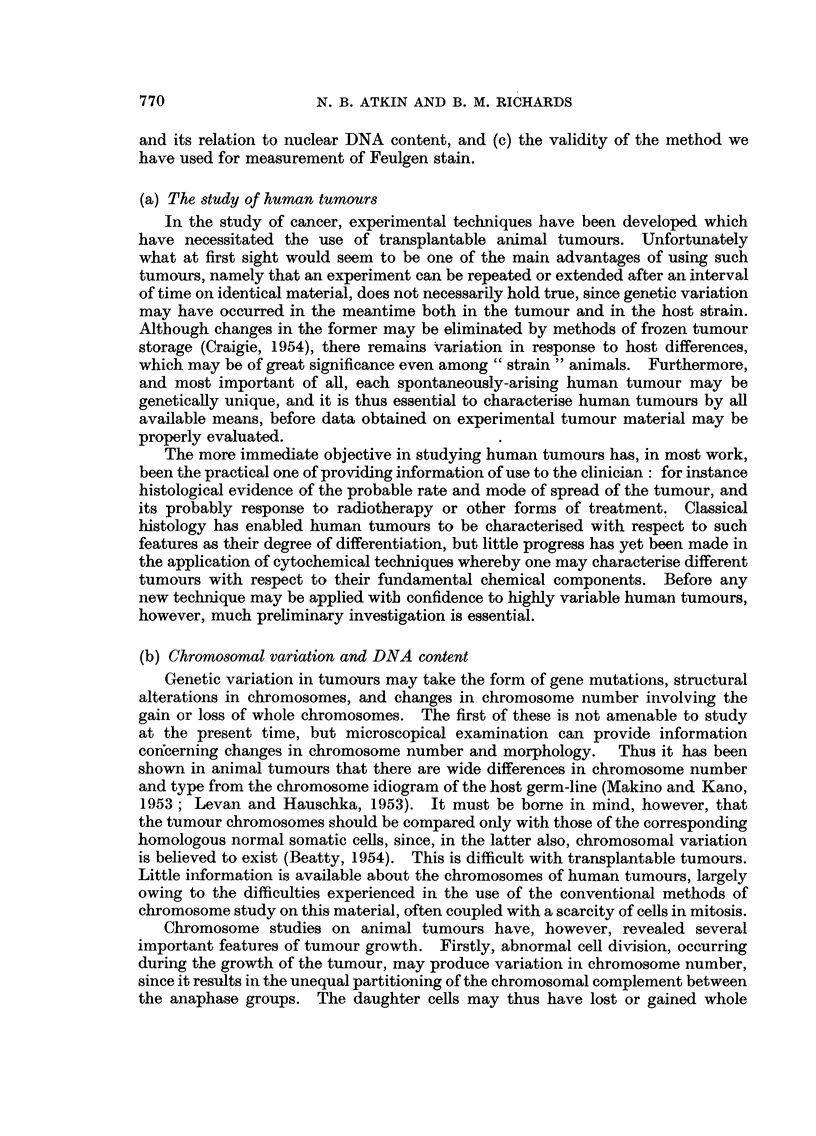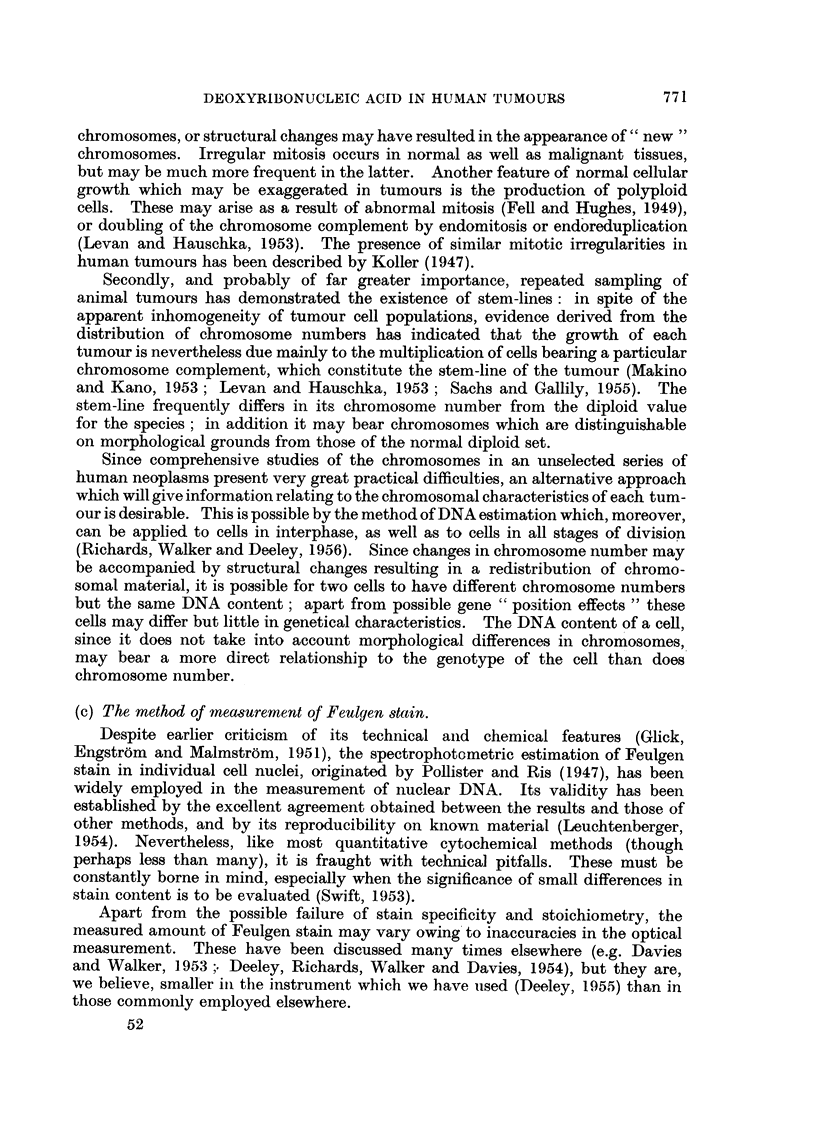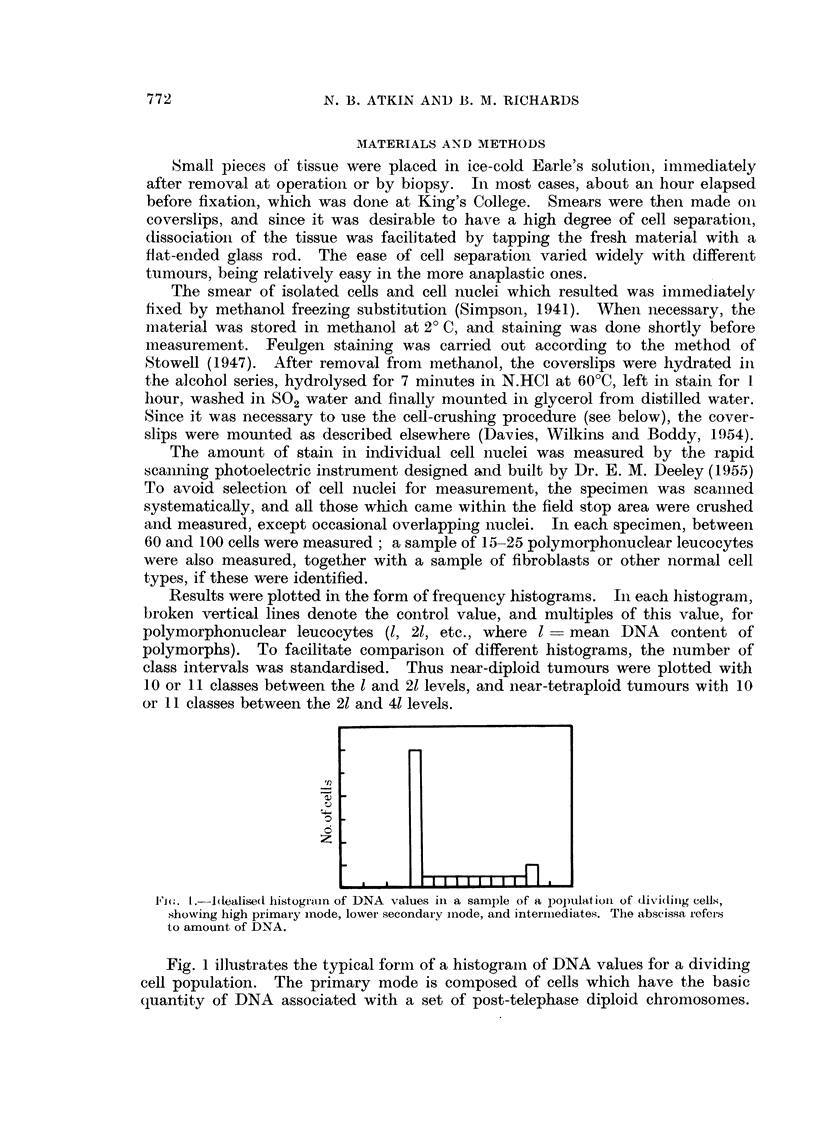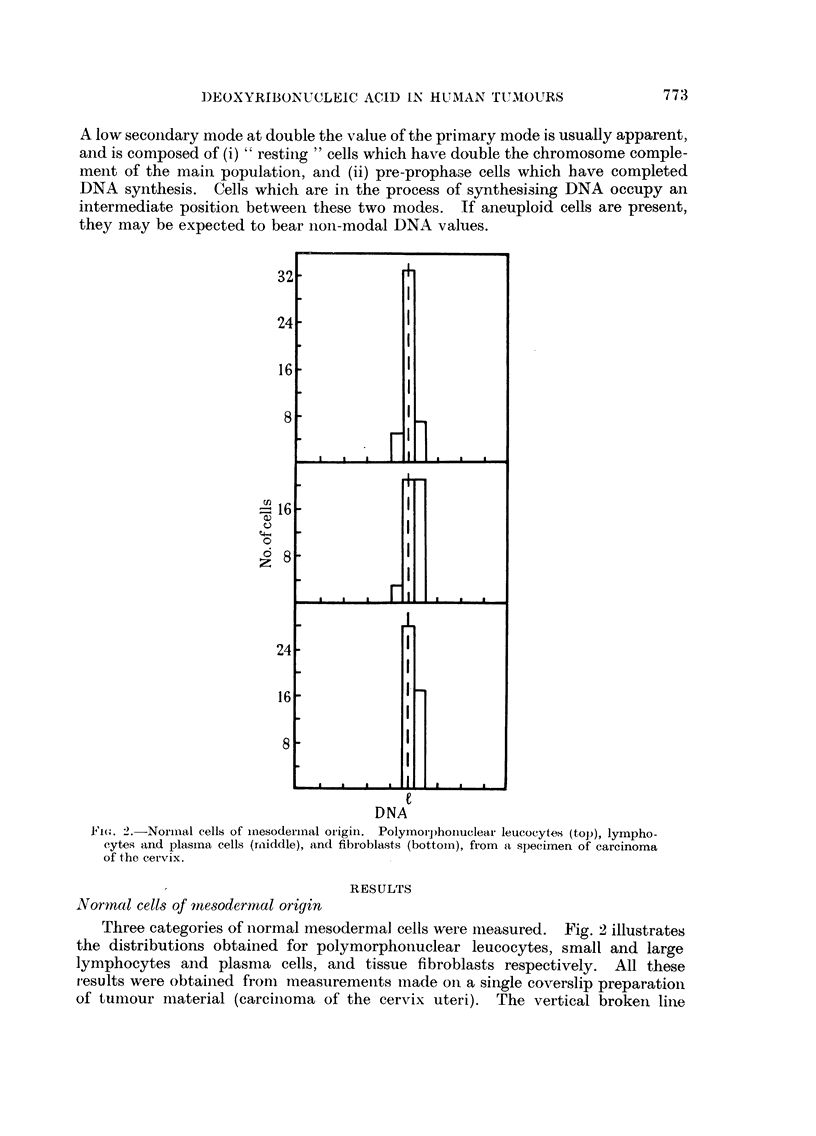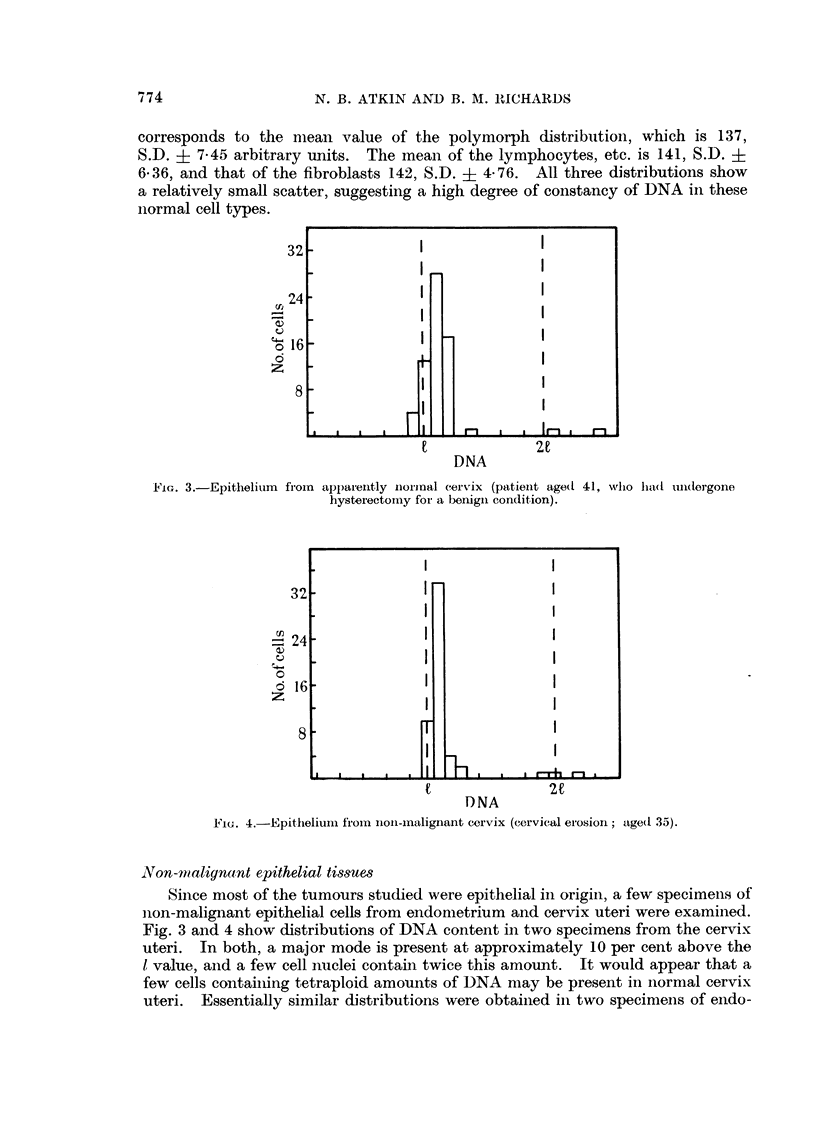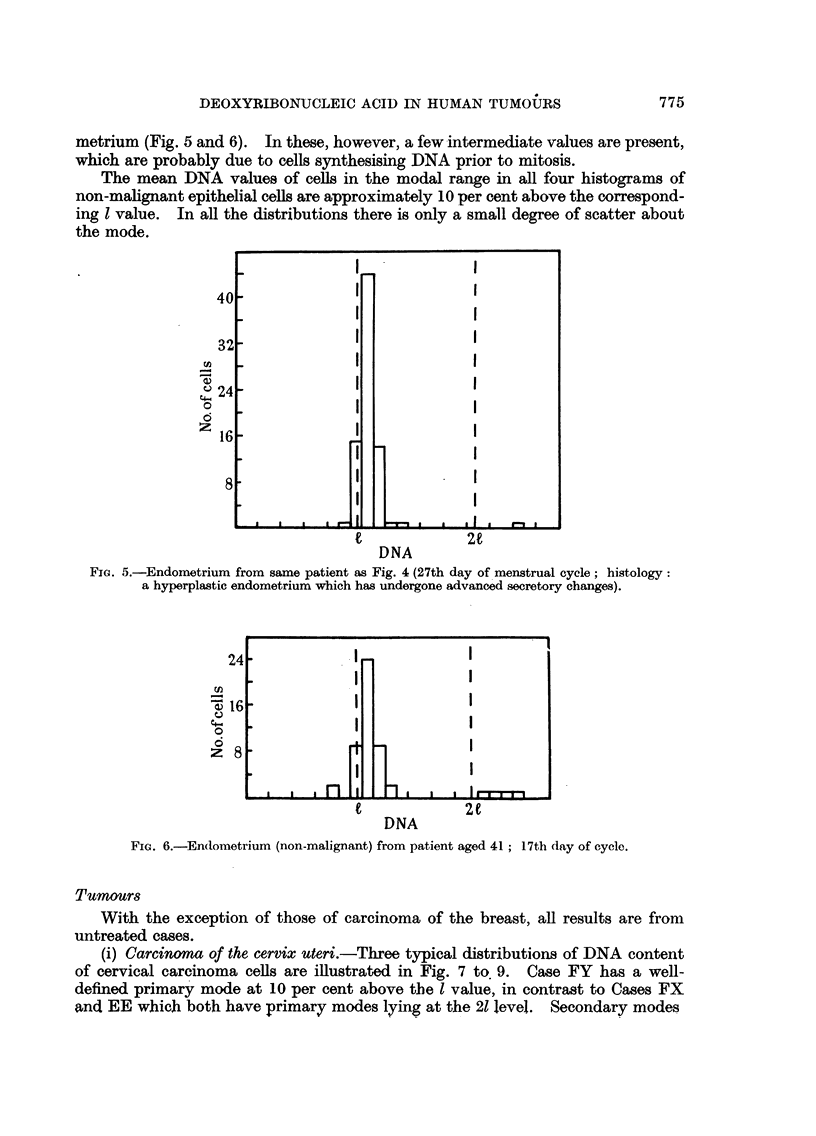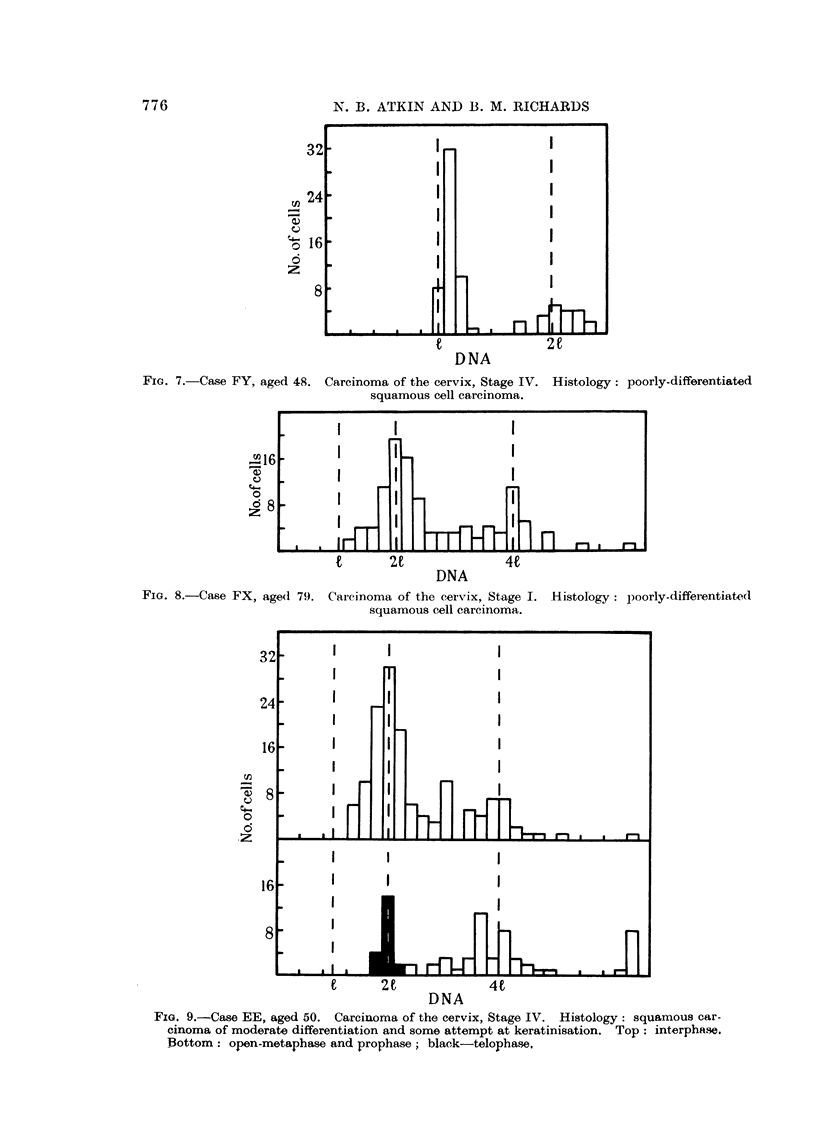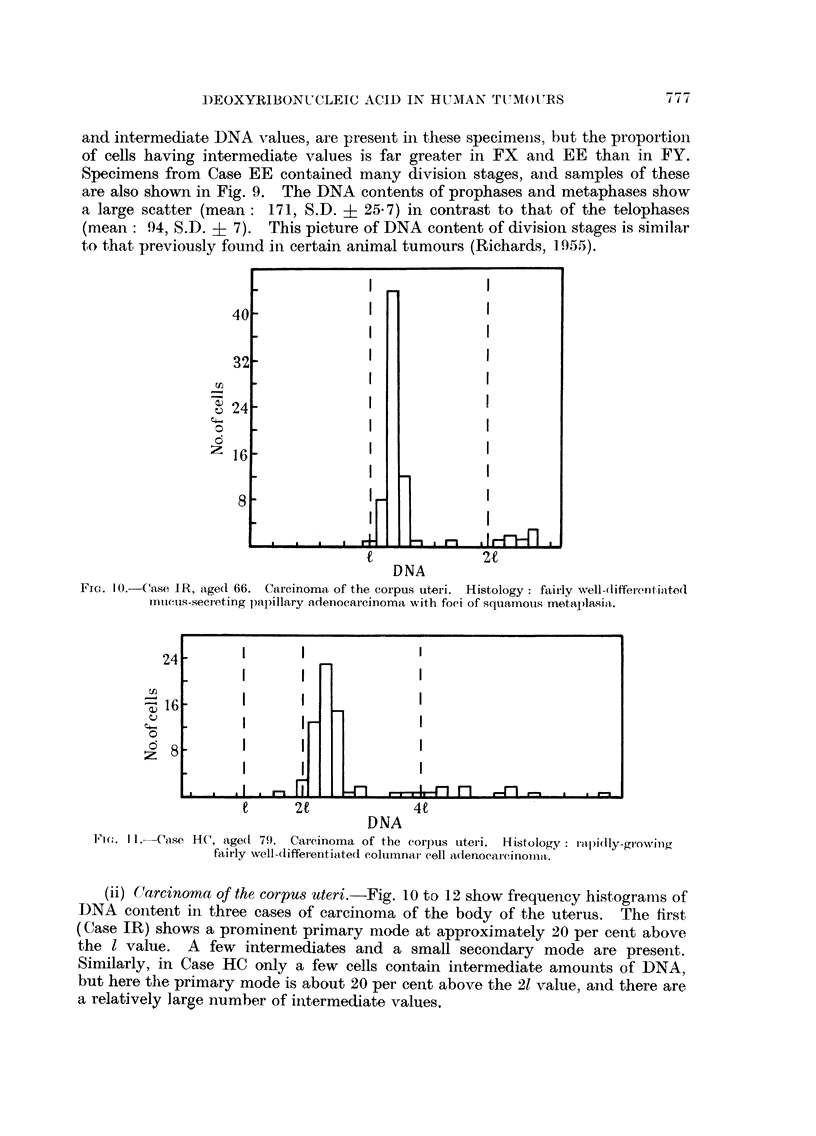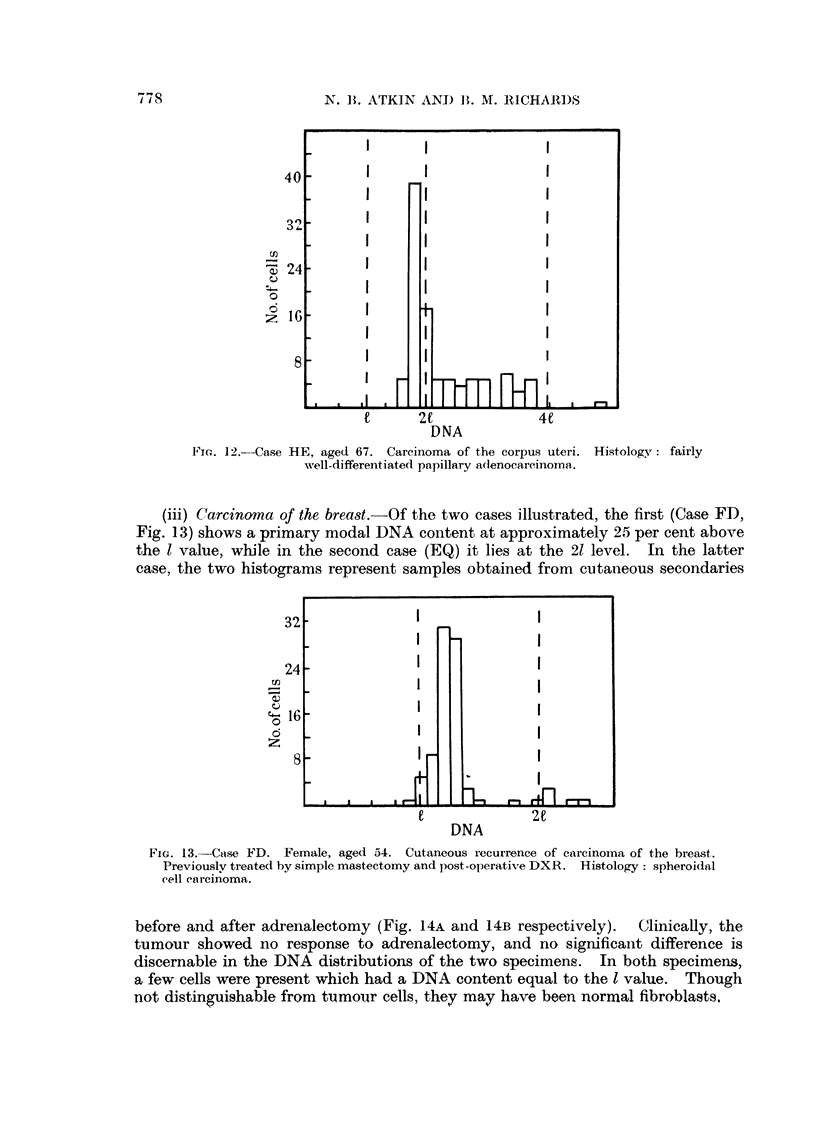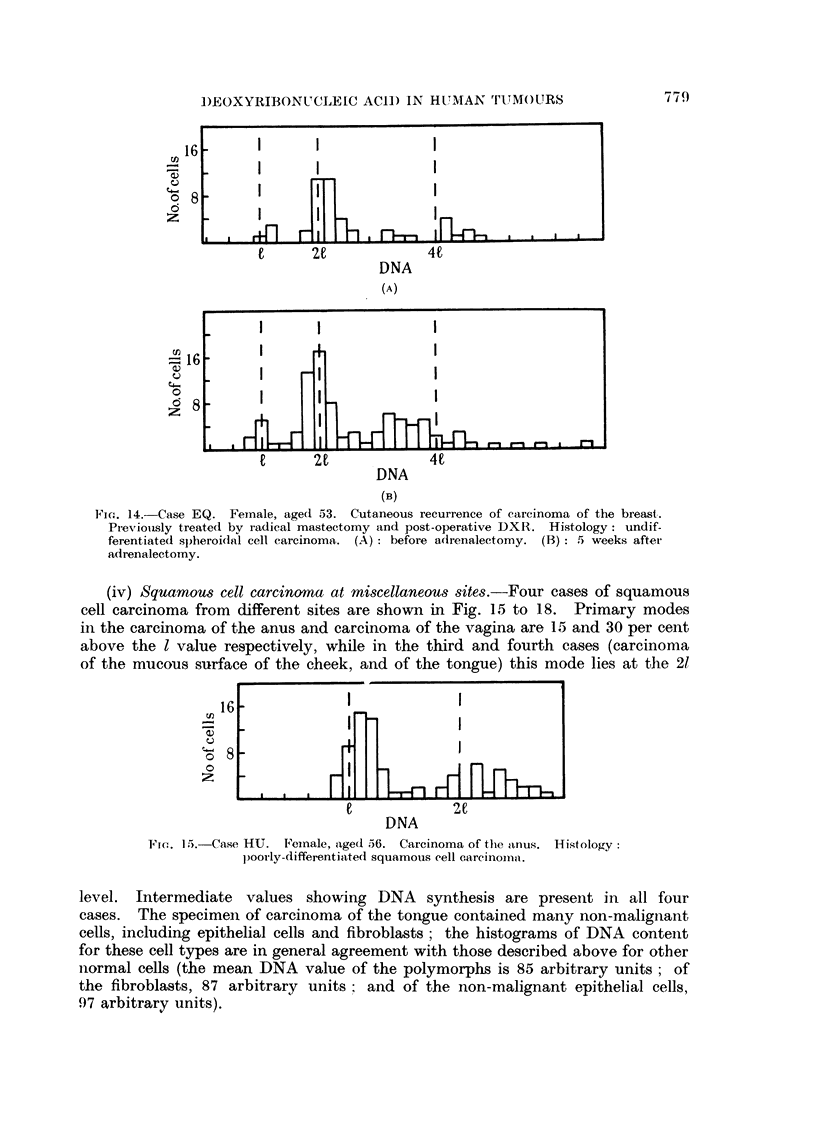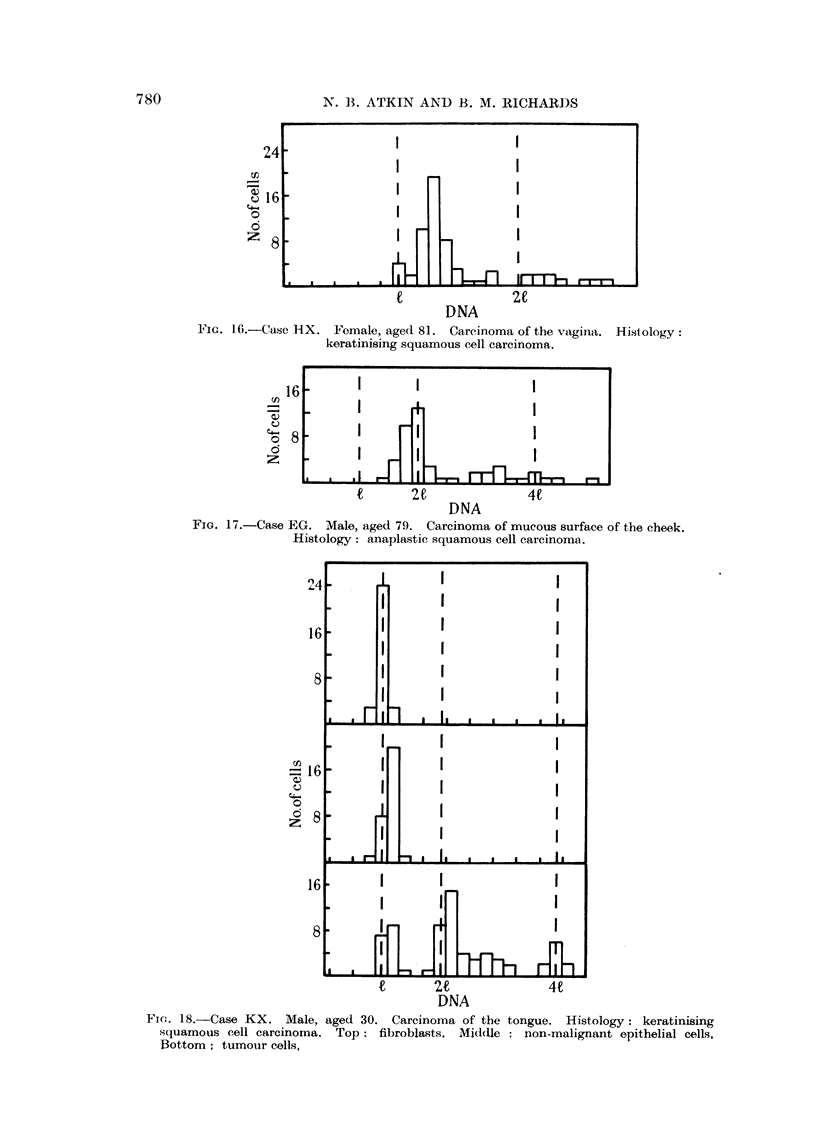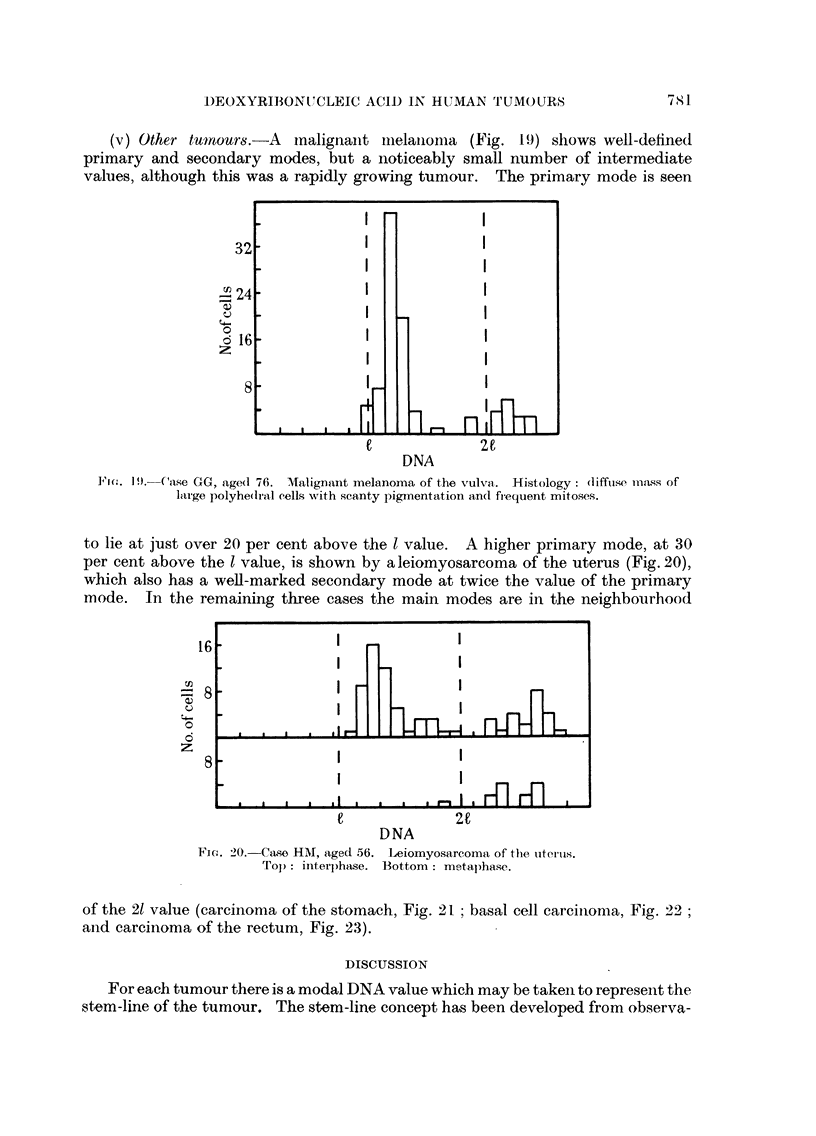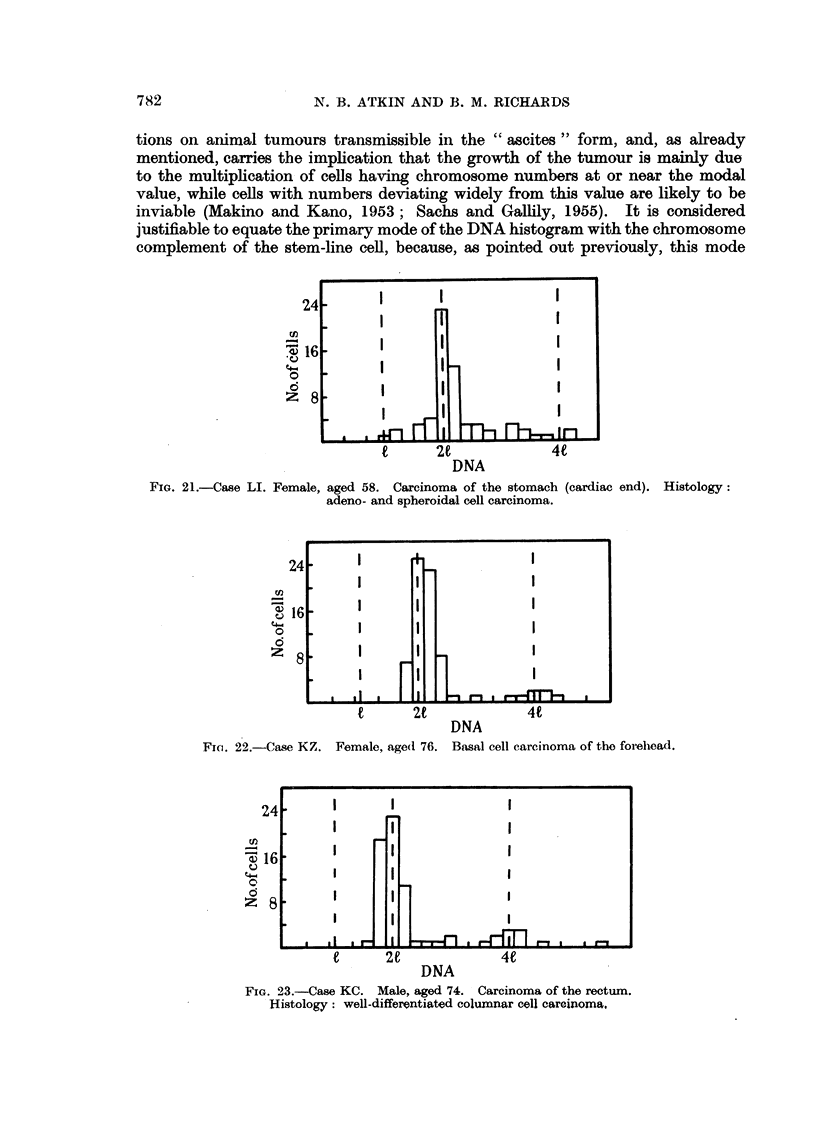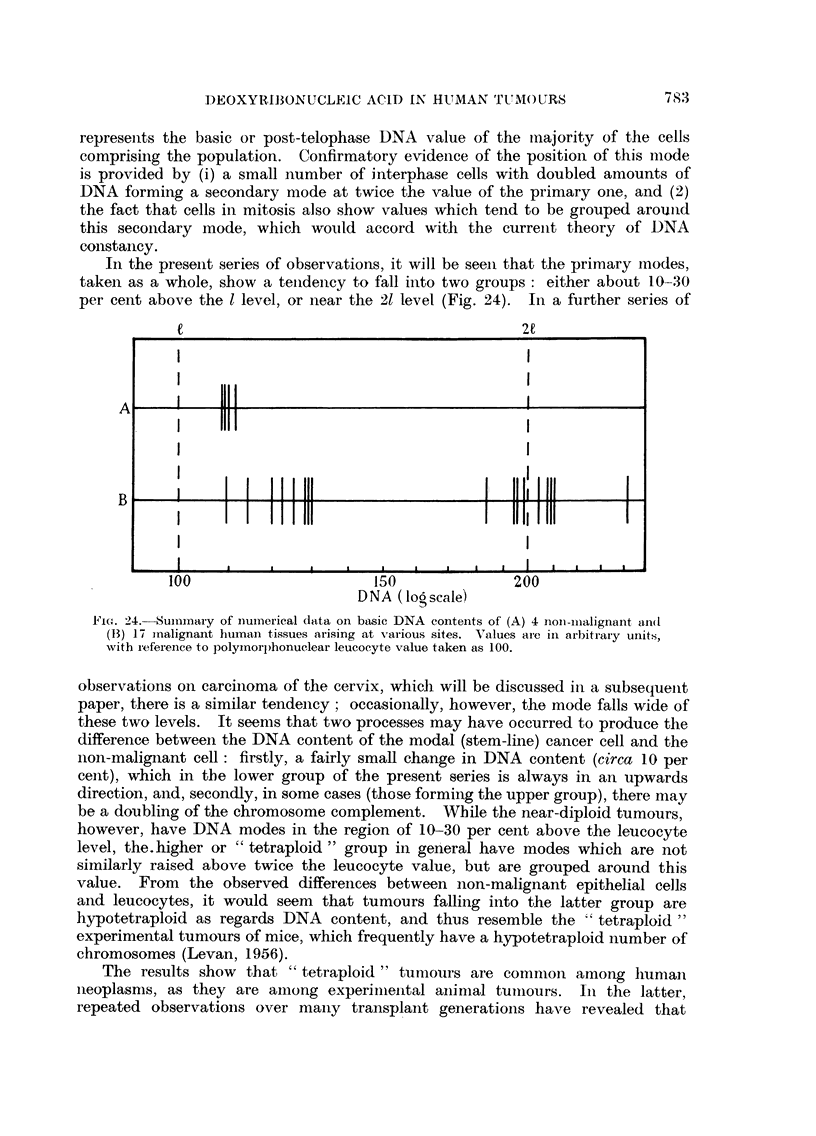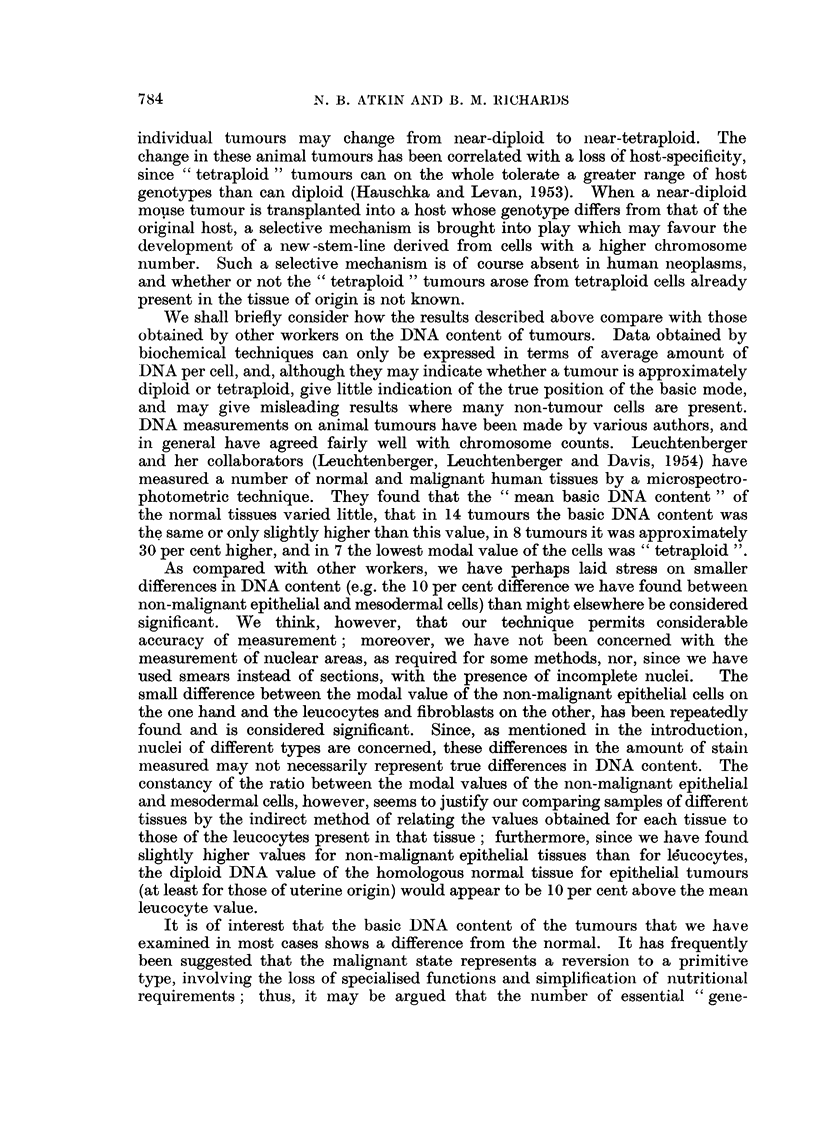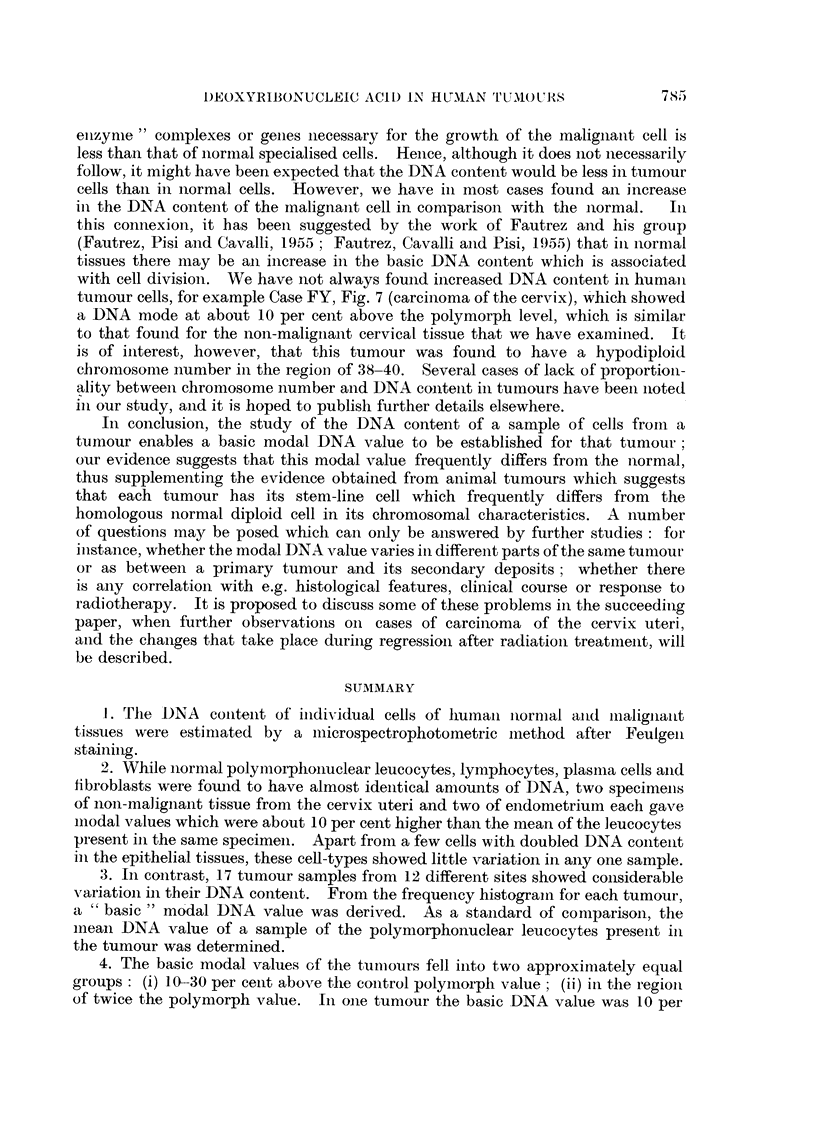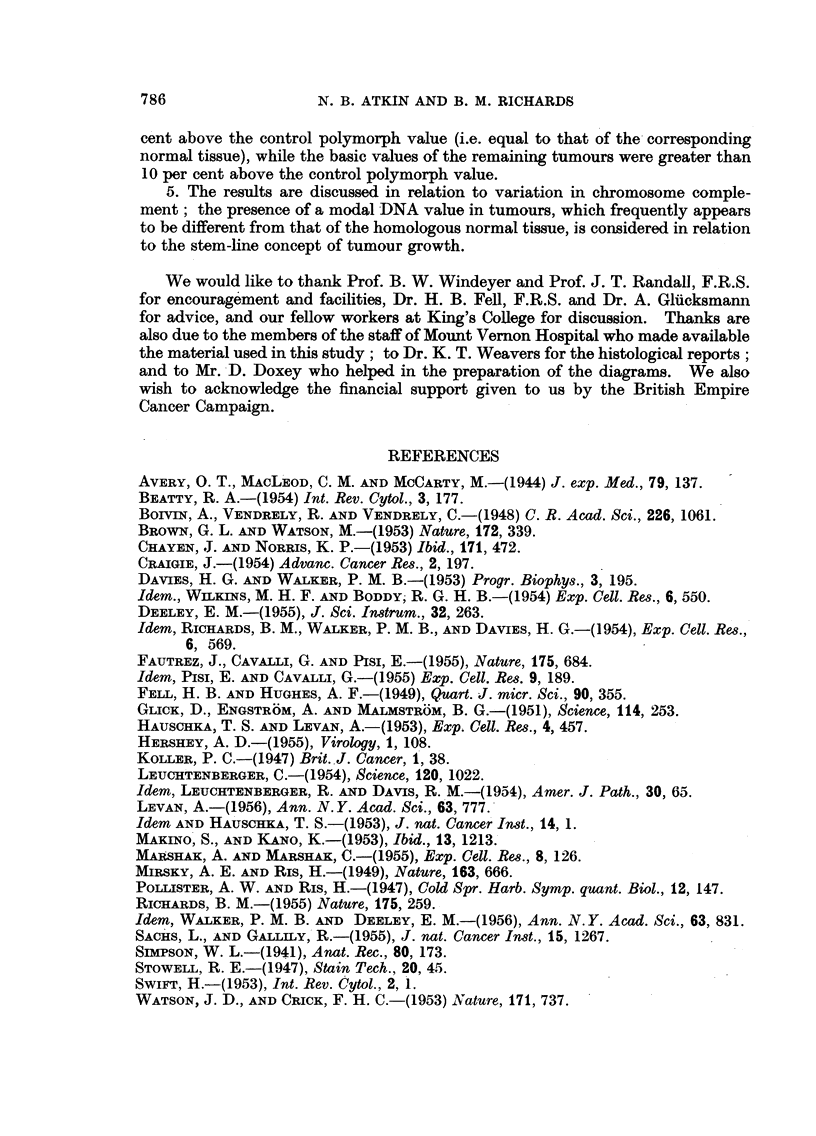# Deoxyribonucleic Acid in Human Tumours as Measured by Microspectrophotometry of Feulgen Stain: A Comparison of Tumours Arising at Different Sites

**DOI:** 10.1038/bjc.1956.94

**Published:** 1956-12

**Authors:** N. B. Atkin, B. M. Richards


### DEOXYRIBONUCLEIC ACID IN HUMAN TUMOURS AS MEASURED BY MICROSPECTROPHOTOMETRY OF FEULGEN STAIN: A COMPARISON OF TUMOURS ARISING AT DIFFERENT SITES

N. B. ATKIN AND B. M. RICHARDS

*From the Department of Cancer Research, Mount Vernon Hospital, Northwood, Middlesex, and the Wheatstone Physics Laboratory, King's College, London, W.C. 2.*

Received for publication August 9, 1956

THE malignant cell differs from the non-malignant cell from which it arose in a number of characteristics which, once established, are transmitted from cell generation to cell generation : the ability of the malignant cell to grow, divide and migrate in an uncontrolled fashion, while it may be modified by environmental factors, must have its basis in transmissible characteristics which differ from those of the normal cell. In recent years, the view that deoxyribonucleic acid (DNA) may be responsible for genetic specificity has received increasing support (Avery, MacLeod and McCarty, 1944 ; Watson and Crick, 1953 ; Hershey, 1955 ; Boivin, Vendrely and Vendrely, 1948 ; Mirsky and Ris, 1949 ; Brown and Watson, 1953). Although the mechanism of the genetic function of DNA has not yet been elucidated, and there are certain anomalous findings which have still to be confirmed (Chayen and Norris, 1953 ; Marshak and Marshak, 1955), it is not unreasonable to assume that the amount of DNA in the individual cell nucleus is proportional to the total content of chromosomal material, and thus is probably related to the gene complement of that nucleus. Therefore, while differences in genetic type may or may not exist between cells containing the same quantity of DNA in their nuclei, it is quite likely that cells containing different amounts of DNA have in fact different genetic characteristics.

In this work, we have measured the amount of Feulgen stain in individual cell nuclei of human tumours. According to existing evidence, the amount of Feulgen stain is proportional to the quantity of DNA in cell nuclei. However, it has not been proved that this relationship holds in all cases ; for instance where different cell types, having different overall chemical compositions, are concerned, it is possible that the amounts of stain may differ, although the same amounts of DNA are present. It is with this reservation in mind that we use the term DNA content here. The purpose of this study is to investigate the DNA content of human tumour cells and to relate these quantitative data to such information as is routinely obtained in clinical, pathological and cytological studies on the same tumours, as well as to similar data obtained from homologous non-malignant tissues. This first paper is concerned with DNA contents of cells from human tumours arising at various sites.

Before presenting the results it is necessary to consider (a) the reasons for obtaining such data from human tumours, (b) chromosomal variation in the growing neoplasm (i.e. structural and numerical changes in the chromosomes)

and its relation to nuclear DNA content, and (c) the validity of the method we have used for measurement of Feulgen stain.

(a) *The study of human tumours*

In the study of cancer, experimental techniques have been developed which have necessitated the use of transplantable animal tumours. Unfortunately what at first sight would seem to be one of the main advantages of using such tumours, namely that an experiment can be repeated or extended after an interval of time on identical material, does not necessarily hold true, since genetic variation may have occurred in the meantime both in the tumour and in the host strain. Although changes in the former may be eliminated by methods of frozen tumour storage (Craigie, 1954), there remains variation in response to host differences, which may be of great significance even among " strain " animals. Furthermore, and most important of all, each spontaneously-arising human tumour may be genetically unique, and it is thus essential to characterise human tumours by all available means, before data obtained on experimental tumour material may be properly evaluated.

The more immediate objective in studying human tumours has, in most work, been the practical one of providing information of use to the clinician : for instance histological evidence of the probable rate and mode of spread of the tumour, and its probably response to radiotherapy or other forms of treatment. Classical histology has enabled human tumours to be characterised with respect to such features as their degree of differentiation, but little progress has yet been made in the application of cytochemical techniques whereby one may characterise different tumours with respect to their fundamental chemical components. Before any new technique may be applied with confidence to highly variable human tumours, however, much preliminary investigation is essential.

(b) *Chromosomal variation and DNA content*

Genetic variation in tumours may take the form of gene mutations, structural alterations in chromosomes, and changes in chromosome number involving the gain or loss of whole chromosomes. The first of these is not amenable to study at the present time, but microscopical examination can provide information concerning changes in chromosome number and morphology. Thus it has been shown in animal tumours that there are wide differences in chromosome number and type from the chromosome idiogram of the host germ-line (Makino and Kano, 1953 ; Levan and Hauschka, 1953). It must be borne in mind, however, that the tumour chromosomes should be compared only with those of the corresponding homologous normal somatic cells, since, in the latter also, chromosomal variation is believed to exist (Beatty, 1954). This is difficult with transplantable tumours. Little information is available about the chromosomes of human tumours, largely owing to the difficulties experienced in the use of the conventional methods of chromosome study on this material, often coupled with a scarcity of cells in mitosis.

Chromosome studies on animal tumours have, however, revealed several important features of tumour growth. Firstly, abnormal cell division, occurring during the growth of the tumour, may produce variation in chromosome number, since it results in the unequal partitioning of the chromosomal complement between the anaphase groups. The daughter cells may thus have lost or gained whole chromosomes, or structural changes may have resulted in the appearance of " new " chromosomes. Irregular mitosis occurs in normal as well as malignant tissues, but may be much more frequent in the latter. Another feature of normal cellular growth which may be exaggerated in tumours is the production of polyploid cells. These may arise as a result of abnormal mitosis (Fell and Hughes, 1949), or doubling of the chromosome complement by endomitosis or endoreduplication (Levan and Hauschka, 1953). The presence of similar mitotic irregularities in human tumours has been described by Koller (1947).

Secondly, and probably of far greater importance, repeated sampling of animal tumours has demonstrated the existence of stem-lines : in spite of the apparent inhomogeneity of tumour cell populations, evidence derived from the distribution of chromosome numbers has indicated that the growth of each tumour is nevertheless due mainly to the multiplication of cells bearing a particular chromosome complement, which constitute the stem-line of the tumour (Makino and Kano, 1953 ; Levan and Hauschka, 1953 ; Sachs and Gallily, 1955). The stem-line frequently differs in its chromosome number from the diploid value for the species ; in addition it may bear chromosomes which are distinguishable on morphological grounds from those of the normal diploid set.

Since comprehensive studies of the chromosomes in an unselected series of human neoplasms present very great practical difficulties, an alternative approach which will give information relating to the chromosomal characteristics of each tumour is desirable. This is possible by the method of DNA estimation which, moreover, can be applied to cells in interphase, as well as to cells in all stages of division (Richards, Walker and Deeley, 1956). Since changes in chromosome number may be accompanied by structural changes resulting in a redistribution of chromosomal material, it is possible for two cells to have different chromosome numbers but the same DNA content ; apart from possible gene " position effects " these cells may differ but little in genetical characteristics. The DNA content of a cell, since it does not take into account morphological differences in chromosomes, may bear a more direct relationship to the genotype of the cell than does chromosome number.

(c) *The method of measurement of Feulgen stain.*

Despite earlier criticism of its technical and chemical features (Glick, Engström and Malmström, 1951), the spectrophotometric estimation of Feulgen stain in individual cell nuclei, originated by Pollister and Ris (1947), has been widely employed in the measurement of nuclear DNA. Its validity has been established by the excellent agreement obtained between the results and those of other methods, and by its reproducibility on known material (Leuchtenberger, 1954). Nevertheless, like most quantitative cytochemical methods (though perhaps less than many), it is fraught with technical pitfalls. These must be constantly borne in mind, especially when the significance of small differences in stain content is to be evaluated (Swift, 1953).

Apart from the possible failure of stain specificity and stoichiometry, the measured amount of Feulgen stain may vary owing to inaccuracies in the optical measurement. These have been discussed many times elsewhere (e.g. Davies and Walker, 1953 ; Deeley, Richards, Walker and Davies, 1954), but they are, we believe, smaller in the instrument which we have used (Deeley, 1955) than in those commonly employed elsewhere.

### MATERIALS AND METHODS

Small pieces of tissue were placed in ice-cold Earle's solution, immediately after removal at operation or by biopsy. In most cases, about an hour elapsed before fixation, which was done at King's College. Smears were then made on coverslips, and since it was desirable to have a high degree of cell separation, dissociation of the tissue was facilitated by tapping the fresh material with a flat-ended glass rod. The ease of cell separation varied widely with different tumours, being relatively easy in the more anaplastic ones.

The smear of isolated cells and cell nuclei which resulted was immediately fixed by methanol freezing substitution (Simpson, 1941). When necessary, the material was stored in methanol at 2° C, and staining was done shortly before measurement. Feulgen staining was carried out according to the method of Stowell (1947). After removal from methanol, the coverslips were hydrated in the alcohol series, hydrolysed for 7 minutes in N.HCl at 60°C, left in stain for 1 hour, washed in $SO_2$ water and finally mounted in glycerol from distilled water. Since it was necessary to use the cell-crushing procedure (see below), the coverslips were mounted as described elsewhere (Davies, Wilkins and Boddy, 1954).

The amount of stain in individual cell nuclei was measured by the rapid scanning photoelectric instrument designed and built by Dr. E. M. Deeley (1955) To avoid selection of cell nuclei for measurement, the specimen was scanned systematically, and all those which came within the field stop area were crushed and measured, except occasional overlapping nuclei. In each specimen, between 60 and 100 cells were measured ; a sample of 15–25 polymorphonuclear leucocytes were also measured, together with a sample of fibroblasts or other normal cell types, if these were identified.

Results were plotted in the form of frequency histograms. In each histogram, broken vertical lines denote the control value, and multiples of this value, for polymorphonuclear leucocytes ($l$, $2l$, etc., where $l$ = mean DNA content of polymorphs). To facilitate comparison of different histograms, the number of class intervals was standardised. Thus near-diploid tumours were plotted with 10 or 11 classes between the $l$ and $2l$ levels, and near-tetraploid tumours with 10 or 11 classes between the $2l$ and $4l$ levels.

FIG. 1.—Idealised histogram of DNA values in a sample of a population of dividing cells, showing high primary mode, lower secondary mode, and intermediates. The abscissa refers to amount of DNA.

Fig. 1 illustrates the typical form of a histogram of DNA values for a dividing cell population. The primary mode is composed of cells which have the basic quantity of DNA associated with a set of post-telephase diploid chromosomes.

A low secondary mode at double the value of the primary mode is usually apparent, and is composed of (i) " resting " cells which have double the chromosome complement of the main population, and (ii) pre-prophase cells which have completed DNA synthesis. Cells which are in the process of synthesising DNA occupy an intermediate position between these two modes. If aneuploid cells are present, they may be expected to bear non-modal DNA values.

FIG. 2.—Normal cells of mesodermal origin. Polymorphonuclear leucocytes (top), lymphocytes and plasma cells (middle), and fibroblasts (bottom), from a specimen of carcinoma of the cervix.

### RESULTS

### *Normal cells of mesodermal origin*

Three categories of normal mesodermal cells were measured. Fig. 2 illustrates the distributions obtained for polymorphonuclear leucocytes, small and large lymphocytes and plasma cells, and tissue fibroblasts respectively. All these results were obtained from measurements made on a single coverslip preparation of tumour material (carcinoma of the cervix uteri). The vertical broken line corresponds to the mean value of the polymorph distribution, which is 137, S.D. ± 7·45 arbitrary units. The mean of the lymphocytes, etc. is 141, S.D. ± 6·36, and that of the fibroblasts 142, S.D. ± 4·76. All three distributions show a relatively small scatter, suggesting a high degree of constancy of DNA in these normal cell types.

FIG. 3.—Epithelium from apparently normal cervix (patient aged 41, who had undergone hysterectomy for a benign condition).

FIG. 4.—Epithelium from non-malignant cervix (cervical erosion ; aged 35).

*Non-malignant epithelial tissues*

Since most of the tumours studied were epithelial in origin, a few specimens of non-malignant epithelial cells from endometrium and cervix uteri were examined. Fig. 3 and 4 show distributions of DNA content in two specimens from the cervix uteri. In both, a major mode is present at approximately 10 per cent above the *l* value, and a few cell nuclei contain twice this amount. It would appear that a few cells containing tetraploid amounts of DNA may be present in normal cervix uteri. Essentially similar distributions were obtained in two specimens of endometrium (Fig. 5 and 6). In these, however, a few intermediate values are present, which are probably due to cells synthesising DNA prior to mitosis.

The mean DNA values of cells in the modal range in all four histograms of non-malignant epithelial cells are approximately 10 per cent above the corresponding $l$ value. In all the distributions there is only a small degree of scatter about the mode.

FIG. 5.—Endometrium from same patient as Fig. 4 (27th day of menstrual cycle ; histology : a hyperplastic endometrium which has undergone advanced secretory changes).

FIG. 6.—Endometrium (non-malignant) from patient aged 41 ; 17th day of cycle.

*Tumours*

With the exception of those of carcinoma of the breast, all results are from untreated cases.

(i) *Carcinoma of the cervix uteri.*—Three typical distributions of DNA content of cervical carcinoma cells are illustrated in Fig. 7 to 9. Case FY has a well-defined primary mode at 10 per cent above the $l$ value, in contrast to Cases FX and EE which both have primary modes lying at the $2l$ level. Secondary modes

FIG. 7.—Case FY, aged 48. Carcinoma of the cervix, Stage IV. Histology : poorly-differentiated squamous cell carcinoma.

FIG. 8.—Case FX, aged 79. Carcinoma of the cervix, Stage I. Histology : poorly-differentiated squamous cell carcinoma.

FIG. 9.—Case EE, aged 50. Carcinoma of the cervix, Stage IV. Histology : squamous carcinoma of moderate differentiation and some attempt at keratinisation. Top : interphase. Bottom : open-metaphase and prophase ; black—telophase.

and intermediate DNA values, are present in these specimens, but the proportion of cells having intermediate values is far greater in FX and EE than in FY. Specimens from Case EE contained many division stages, and samples of these are also shown in Fig. 9. The DNA contents of prophases and metaphases show a large scatter (mean : 171, S.D. ± 25·7) in contrast to that of the telophases (mean : 94, S.D. ± 7). This picture of DNA content of division stages is similar to that previously found in certain animal tumours (Richards, 1955).

Fig. 10.—Case IR, aged 66. Carcinoma of the corpus uteri. Histology : fairly well-differentiated mucus-secreting papillary adenocarcinoma with foci of squamous metaplasia.

Fig. 11.—Case HC, aged 79. Carcinoma of the corpus uteri. Histology : rapidly-growing fairly well-differentiated columnar cell adenocarcinoma.

(ii) *Carcinoma of the corpus uteri.*—Fig. 10 to 12 show frequency histograms of DNA content in three cases of carcinoma of the body of the uterus. The first (Case IR) shows a prominent primary mode at approximately 20 per cent above the $l$ value. A few intermediates and a small secondary mode are present. Similarly, in Case HC only a few cells contain intermediate amounts of DNA, but here the primary mode is about 20 per cent above the $2l$ value, and there are a relatively large number of intermediate values.

FIG. 12.—Case HE, aged 67. Carcinoma of the corpus uteri. Histology : fairly well-differentiated papillary adenocarcinoma.

(iii) *Carcinoma of the breast.*—Of the two cases illustrated, the first (Case FD, Fig. 13) shows a primary modal DNA content at approximately 25 per cent above the $l$ value, while in the second case (EQ) it lies at the $2l$ level. In the latter case, the two histograms represent samples obtained from cutaneous secondaries

FIG. 13.—Case FD. Female, aged 54. Cutaneous recurrence of carcinoma of the breast. Previously treated by simple mastectomy and post-operative DXR. Histology : spheroidal cell carcinoma.

before and after adrenalectomy (Fig. 14A and 14B respectively). Clinically, the tumour showed no response to adrenalectomy, and no significant difference is discernable in the DNA distributions of the two specimens. In both specimens, a few cells were present which had a DNA content equal to the $l$ value. Though not distinguishable from tumour cells, they may have been normal fibroblasts.

FIG. 14.—Case EQ. Female, aged 53. Cutaneous recurrence of carcinoma of the breast. Previously treated by radical mastectomy and post-operative DXR. Histology: undifferentiated spheroidal cell carcinoma. (A): before adrenalectomy. (B): 5 weeks after adrenalectomy.

(iv) *Squamous cell carcinoma at miscellaneous sites.*—Four cases of squamous cell carcinoma from different sites are shown in Fig. 15 to 18. Primary modes in the carcinoma of the anus and carcinoma of the vagina are 15 and 30 per cent above the $l$ value respectively, while in the third and fourth cases (carcinoma of the mucous surface of the cheek, and of the tongue) this mode lies at the $2l$

FIG. 15.—Case HU. Female, aged 56. Carcinoma of the anus. Histology: poorly-differentiated squamous cell carcinoma.

level. Intermediate values showing DNA synthesis are present in all four cases. The specimen of carcinoma of the tongue contained many non-malignant cells, including epithelial cells and fibroblasts; the histograms of DNA content for these cell types are in general agreement with those described above for other normal cells (the mean DNA value of the polymorphs is 85 arbitrary units; of the fibroblasts, 87 arbitrary units; and of the non-malignant epithelial cells, 97 arbitrary units).

FIG. 16.—Case HX. Female, aged 81. Carcinoma of the vagina. Histology : keratinising squamous cell carcinoma.

FIG. 17.—Case EG. Male, aged 79. Carcinoma of mucous surface of the cheek. Histology : anaplastic squamous cell carcinoma.

FIG. 18.—Case KX. Male, aged 30. Carcinoma of the tongue. Histology : keratinising squamous cell carcinoma. Top : fibroblasts. Middle : non-malignant epithelial cells. Bottom ; tumour cells.

(v) *Other tumours.*—A malignant melanoma (Fig. 19) shows well-defined primary and secondary modes, but a noticeably small number of intermediate values, although this was a rapidly growing tumour. The primary mode is seen

FIG. 19.—Case GG, aged 76. Malignant melanoma of the vulva. Histology : diffuse mass of large polyhedral cells with scanty pigmentation and frequent mitoses.

to lie at just over 20 per cent above the $l$ value. A higher primary mode, at 30 per cent above the $l$ value, is shown by a leiomyosarcoma of the uterus (Fig. 20), which also has a well-marked secondary mode at twice the value of the primary mode. In the remaining three cases the main modes are in the neighbourhood

FIG. 20.—Case HM, aged 56. Leiomyosarcoma of the uterus. Top : interphase. Bottom : metaphase.

of the $2l$ value (carcinoma of the stomach, Fig. 21 ; basal cell carcinoma, Fig. 22 ; and carcinoma of the rectum, Fig. 23).

### DISCUSSION

For each tumour there is a modal DNA value which may be taken to represent the stem-line of the tumour. The stem-line concept has been developed from observations on animal tumours transmissible in the " ascites " form, and, as already mentioned, carries the implication that the growth of the tumour is mainly due to the multiplication of cells having chromosome numbers at or near the modal value, while cells with numbers deviating widely from this value are likely to be inviable (Makino and Kano, 1953 ; Sachs and Gallily, 1955). It is considered justifiable to equate the primary mode of the DNA histogram with the chromosome complement of the stem-line cell, because, as pointed out previously, this mode

FIG. 21.—Case LI. Female, aged 58. Carcinoma of the stomach (cardiac end). Histology : adeno- and spheroidal cell carcinoma.

FIG. 22.—Case KZ. Female, aged 76. Basal cell carcinoma of the forehead.

FIG. 23.—Case KC. Male, aged 74. Carcinoma of the rectum. Histology : well-differentiated columnar cell carcinoma.

represents the basic or post-telophase DNA value of the majority of the cells comprising the population. Confirmatory evidence of the position of this mode is provided by (i) a small number of interphase cells with doubled amounts of DNA forming a secondary mode at twice the value of the primary one, and (2) the fact that cells in mitosis also show values which tend to be grouped around this secondary mode, which would accord with the current theory of DNA constancy.

In the present series of observations, it will be seen that the primary modes, taken as a whole, show a tendency to fall into two groups : either about 10–30 per cent above the *l* level, or near the 2*l* level (Fig. 24). In a further series of

FIG. 24.—Summary of numerical data on basic DNA contents of (A) 4 non-malignant and (B) 17 malignant human tissues arising at various sites. Values are in arbitrary units, with reference to polymorphonuclear leucocyte value taken as 100.

observations on carcinoma of the cervix, which will be discussed in a subsequent paper, there is a similar tendency ; occasionally, however, the mode falls wide of these two levels. It seems that two processes may have occurred to produce the difference between the DNA content of the modal (stem-line) cancer cell and the non-malignant cell : firstly, a fairly small change in DNA content (*circa* 10 per cent), which in the lower group of the present series is always in an upwards direction, and, secondly, in some cases (those forming the upper group), there may be a doubling of the chromosome complement. While the near-diploid tumours, however, have DNA modes in the region of 10–30 per cent above the leucocyte level, the higher or " tetraploid " group in general have modes which are not similarly raised above twice the leucocyte value, but are grouped around this value. From the observed differences between non-malignant epithelial cells and leucocytes, it would seem that tumours falling into the latter group are hypotetraploid as regards DNA content, and thus resemble the " tetraploid " experimental tumours of mice, which frequently have a hypotetraploid number of chromosomes (Levan, 1956).

The results show that " tetraploid " tumours are common among human neoplasms, as they are among experimental animal tumours. In the latter, repeated observations over many transplant generations have revealed that individual tumours may change from near-diploid to near-tetraploid. The change in these animal tumours has been correlated with a loss of host-specificity, since " tetraploid " tumours can on the whole tolerate a greater range of host genotypes than can diploid (Hauschka and Levan, 1953). When a near-diploid mouse tumour is transplanted into a host whose genotype differs from that of the original host, a selective mechanism is brought into play which may favour the development of a new-stem-line derived from cells with a higher chromosome number. Such a selective mechanism is of course absent in human neoplasms, and whether or not the " tetraploid " tumours arose from tetraploid cells already present in the tissue of origin is not known.

We shall briefly consider how the results described above compare with those obtained by other workers on the DNA content of tumours. Data obtained by biochemical techniques can only be expressed in terms of average amount of DNA per cell, and, although they may indicate whether a tumour is approximately diploid or tetraploid, give little indication of the true position of the basic mode, and may give misleading results where many non-tumour cells are present. DNA measurements on animal tumours have been made by various authors, and in general have agreed fairly well with chromosome counts. Leuchtenberger and her collaborators (Leuchtenberger, Leuchtenberger and Davis, 1954) have measured a number of normal and malignant human tissues by a microspectrophotometric technique. They found that the " mean basic DNA content " of the normal tissues varied little, that in 14 tumours the basic DNA content was the same or only slightly higher than this value, in 8 tumours it was approximately 30 per cent higher, and in 7 the lowest modal value of the cells was " tetraploid ".

As compared with other workers, we have perhaps laid stress on smaller differences in DNA content (e.g. the 10 per cent difference we have found between non-malignant epithelial and mesodermal cells) than might elsewhere be considered significant. We think, however, that our technique permits considerable accuracy of measurement ; moreover, we have not been concerned with the measurement of nuclear areas, as required for some methods, nor, since we have used smears instead of sections, with the presence of incomplete nuclei. The small difference between the modal value of the non-malignant epithelial cells on the one hand and the leucocytes and fibroblasts on the other, has been repeatedly found and is considered significant. Since, as mentioned in the introduction, nuclei of different types are concerned, these differences in the amount of stain measured may not necessarily represent true differences in DNA content. The constancy of the ratio between the modal values of the non-malignant epithelial and mesodermal cells, however, seems to justify our comparing samples of different tissues by the indirect method of relating the values obtained for each tissue to those of the leucocytes present in that tissue ; furthermore, since we have found slightly higher values for non-malignant epithelial tissues than for leucocytes, the diploid DNA value of the homologous normal tissue for epithelial tumours (at least for those of uterine origin) would appear to be 10 per cent above the mean leucocyte value.

It is of interest that the basic DNA content of the tumours that we have examined in most cases shows a difference from the normal. It has frequently been suggested that the malignant state represents a reversion to a primitive type, involving the loss of specialised functions and simplification of nutritional requirements ; thus, it may be argued that the number of essential " geneenzyme " complexes or genes necessary for the growth of the malignant cell is less than that of normal specialised cells. Hence, although it does not necessarily follow, it might have been expected that the DNA content would be less in tumour cells than in normal cells. However, we have in most cases found an increase in the DNA content of the malignant cell in comparison with the normal. In this connexion, it has been suggested by the work of Fautrez and his group (Fautrez, Pisi and Cavalli, 1955 ; Fautrez, Cavalli and Pisi, 1955) that in normal tissues there may be an increase in the basic DNA content which is associated with cell division. We have not always found increased DNA content in human tumour cells, for example Case FY, Fig. 7 (carcinoma of the cervix), which showed a DNA mode at about 10 per cent above the polymorph level, which is similar to that found for the non-malignant cervical tissue that we have examined. It is of interest, however, that this tumour was found to have a hypodiploid chromosome number in the region of 38–40. Several cases of lack of proportionality between chromosome number and DNA content in tumours have been noted in our study, and it is hoped to publish further details elsewhere.

In conclusion, the study of the DNA content of a sample of cells from a tumour enables a basic modal DNA value to be established for that tumour ; our evidence suggests that this modal value frequently differs from the normal, thus supplementing the evidence obtained from animal tumours which suggests that each tumour has its stem-line cell which frequently differs from the homologous normal diploid cell in its chromosomal characteristics. A number of questions may be posed which can only be answered by further studies : for instance, whether the modal DNA value varies in different parts of the same tumour or as between a primary tumour and its secondary deposits ; whether there is any correlation with e.g. histological features, clinical course or response to radiotherapy. It is proposed to discuss some of these problems in the succeeding paper, when further observations on cases of carcinoma of the cervix uteri, and the changes that take place during regression after radiation treatment, will be described.

### SUMMARY

1. The DNA content of individual cells of human normal and malignant tissues were estimated by a microspectrophotometric method after Feulgen staining.

2. While normal polymorphonuclear leucocytes, lymphocytes, plasma cells and fibroblasts were found to have almost identical amounts of DNA, two specimens of non-malignant tissue from the cervix uteri and two of endometrium each gave modal values which were about 10 per cent higher than the mean of the leucocytes present in the same specimen. Apart from a few cells with doubled DNA content in the epithelial tissues, these cell-types showed little variation in any one sample.

3. In contrast, 17 tumour samples from 12 different sites showed considerable variation in their DNA content. From the frequency histogram for each tumour, a " basic " modal DNA value was derived. As a standard of comparison, the mean DNA value of a sample of the polymorphonuclear leucocytes present in the tumour was determined.

4. The basic modal values of the tumours fell into two approximately equal groups : (i) 10–30 per cent above the control polymorph value ; (ii) in the region of twice the polymorph value. In one tumour the basic DNA value was 10 per cent above the control polymorph value (i.e. equal to that of the corresponding normal tissue), while the basic values of the remaining tumours were greater than 10 per cent above the control polymorph value.

5. The results are discussed in relation to variation in chromosome complement ; the presence of a modal DNA value in tumours, which frequently appears to be different from that of the homologous normal tissue, is considered in relation to the stem-line concept of tumour growth.

We would like to thank Prof. B. W. Windeyer and Prof. J. T. Randall, F.R.S. for encouragement and facilities, Dr. H. B. Fell, F.R.S. and Dr. A. Glücksmann for advice, and our fellow workers at King's College for discussion. Thanks are also due to the members of the staff of Mount Vernon Hospital who made available the material used in this study ; to Dr. K. T. Weavers for the histological reports ; and to Mr. D. Doxey who helped in the preparation of the diagrams. We also wish to acknowledge the financial support given to us by the British Empire Cancer Campaign.

### REFERENCES

AVERY, O. T., MACLEOD, C. M. AND MCCARTY, M.—(1944) *J. exp. Med.*, **79**, 137.
BEATTY, R. A.—(1954) *Int. Rev. Cytol.*, **3**, 177.
BOIVIN, A., VENDRELY, R. AND VENDRELY, C.—(1948) *C. R. Acad. Sci.*, **226**, 1061.
BROWN, G. L. AND WATSON, M.—(1953) *Nature*, **172**, 339.
CHAYEN, J. AND NORRIS, K. P.—(1953) *Ibid.*, **171**, 472.
CRAIGIE, J.—(1954) *Advanc. Cancer Res.*, **2**, 197.
DAVIES, H. G. AND WALKER, P. M. B.—(1953) *Progr. Biophys.*, **3**, 195.
*Idem.*, WILKINS, M. H. F. AND BODDY, R. G. H. B.—(1954) *Exp. Cell. Res.*, **6**, 550.
DEELEY, E. M.—(1955), *J. Sci. Instrum.*, **32**, 263.
*Idem*, RICHARDS, B. M., WALKER, P. M. B., AND DAVIES, H. G.—(1954), *Exp. Cell. Res.*, **6**, 569.
FAUTREZ, J., CAVALLI, G. AND PISI, E.—(1955), *Nature*, **175**, 684.
*Idem*, PISI, E. AND CAVALLI, G.—(1955) *Exp. Cell. Res.* **9**, 189.
FELL, H. B. AND HUGHES, A. F.—(1949), *Quart. J. micr. Sci.*, **90**, 355.
GLICK, D., ENGSTRÖM, A. AND MALMSTRÖM, B. G.—(1951), *Science*, **114**, 253.
HAUSCHKA, T. S. AND LEVAN, A.—(1953), *Exp. Cell. Res.*, **4**, 457.
HERSHEY, A. D.—(1955), *Virology*, **1**, 108.
KOLLER, P. C.—(1947) *Brit. J. Cancer*, **1**, 38.
LEUCHTENBERGER, C.—(1954), *Science*, **120**, 1022.
*Idem*, LEUCHTENBERGER, R. AND DAVIS, R. M.—(1954), *Amer. J. Path.*, **30**, 65.
LEVAN, A.—(1956), *Ann. N.Y. Acad. Sci.*, **63**, 777.
*Idem* AND HAUSCHKA, T. S.—(1953), *J. nat. Cancer Inst.*, **14**, 1.
MAKINO, S., AND KANO, K.—(1953), *Ibid.*, **13**, 1213.
MARSHAK, A. AND MARSHAK, C.—(1955), *Exp. Cell. Res.*, **8**, 126.
MIRSKY, A. E. AND RIS, H.—(1949), *Nature*, **163**, 666.
POLLISTER, A. W. AND RIS, H.—(1947), *Cold Spr. Harb. Symp. quant. Biol.*, **12**, 147.
RICHARDS, B. M.—(1955) *Nature*, **175**, 259.
*Idem*, WALKER, P. M. B. AND DEELEY, E. M.—(1956), *Ann. N.Y. Acad. Sci.*, **63**, 831.
SACHS, L., AND GALLILY, R.—(1955), *J. nat. Cancer Inst.*, **15**, 1267.
SIMPSON, W. L.—(1941), *Anat. Rec.*, **80**, 173.
STOWELL, R. E.—(1947), *Stain Tech.*, **20**, 45.
SWIFT, H.—(1953), *Int. Rev. Cytol.*, **2**, 1.
WATSON, J. D., AND CRICK, F. H. C.—(1953) *Nature*, **171**, 737.